# Chemotherapy-induced intestinal epithelial damage directly promotes galectin-9-driven modulation of T cell behavior

**DOI:** 10.1016/j.isci.2024.110072

**Published:** 2024-05-22

**Authors:** Suze A. Jansen, Alessandro Cutilli, Coco de Koning, Marliek van Hoesel, Cynthia L. Frederiks, Leire Saiz Sierra, Stefan Nierkens, Michal Mokry, Edward E.S. Nieuwenhuis, Alan M. Hanash, Enric Mocholi, Paul J. Coffer, Caroline A. Lindemans

**Affiliations:** 1 Division of Pediatrics, University Medical Center Utrecht, Utrecht 3584GX, the Netherlands; 2 Princess Máxima Center for Pediatric Oncology, Utrecht 3584CS, the Netherlands; 3 Regenerative Medicine Center, University Medical Center Utrecht, Utrecht 3584CT, the Netherlands; 4 Center of Molecular Medicine, University Medical Center Utrecht, Utrecht 3584CG, the Netherlands; 5 Center for Translational Immunology, University Medical Center Utrecht, 3584GX Utrecht, the Netherlands; 6 Department of Cardiology, University Medical Center Utrecht, Utrecht 3584GX, the Netherlands; 7 University College Roosevelt, Utrecht University, Middelburg 4331CB, the Netherlands; 8 Departments of Medicine and Human Oncology & Pathogenesis Program, Memorial Sloan Kettering Cancer Center and Weill Cornell Medical College, New York, NY 10065, USA

**Keywords:** Molecular biology, Immunology, Stem cells research, Cancer

## Abstract

The intestine is vulnerable to chemotherapy-induced damage due to the high rate of intestinal epithelial cell (IEC) proliferation. We have developed a human intestinal organoid-based 3D model system to study the direct effect of chemotherapy-induced IEC damage on T cell behavior. Exposure of intestinal organoids to busulfan, fludarabine, and clofarabine induced damage-related responses affecting both the capacity to regenerate and transcriptional reprogramming. In *ex vivo* co-culture assays, prior intestinal organoid damage resulted in increased T cell activation, proliferation, and migration. We identified galectin-9 (Gal-9) as a key molecule released by damaged organoids. The use of anti-Gal-9 blocking antibodies or CRISPR/Cas9-mediated Gal-9 knock-out prevented intestinal organoid damage-induced T cell proliferation, interferon-gamma release, and migration. Increased levels of Gal-9 were found early after HSCT chemotherapeutic conditioning in the plasma of patients who later developed acute GVHD. Taken together, chemotherapy-induced intestinal damage can influence T cell behavior in a Gal-9-dependent manner which may provide novel strategies for therapeutic intervention.

## Highlights

• Chemotherapy-induced intestinal epithelial damage can be modeled with organoids
• 3D-coculture model allows evaluation of epithelial damage on T cell homeostasis
• Chemotherapy-induced epithelial damage modulates T cell activation and migration
• Galectin-9 promotes T cell activation and migration in response to epithelial damage

## Graphical abstract

**Figure:**
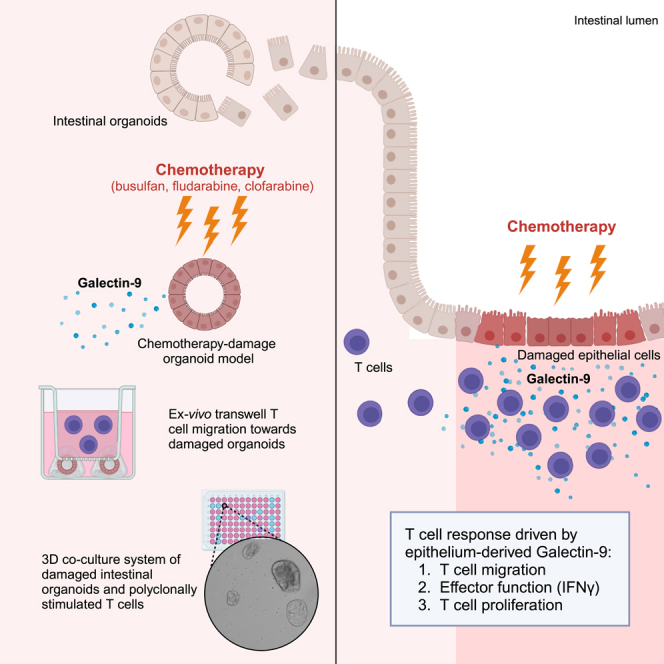

## Introduction

The intestinal epithelium comprises a variety of secretory and absorptive intestinal epithelial cell (IEC) types that derive from the intestinal stem cells (ISCs) at the bottom of intestinal epithelial crypts. The intestinal epithelium forms a physical barrier between the gut lumen and milieu interior, and as such provides the first layer of defense against harmful luminal components and pathogens.^1^ Damage to the intestinal epithelium has been linked to immune activation with a T cell component in multiple disease settings, including inflammatory bowel disease (IBD), celiac disease, and acute intestinal graft-versus-host disease (GVHD) after allogeneic hematopoietic stem cell transplantation (HSCT).^1^^,^^2^^,^^3^^,^^4^^,^^5^^,^^6^^,^^7^^,^^8^ How epithelial damage may directly influence T cell behavior still needs to be completely elucidated.

Mechanisms of T cell activation after epithelial damage are thought to involve innate immune cell activation including neutrophils, monocytes, and macrophages, leading to migration of T cells, and both local antigen presentation, as well as antigen presentation by professional antigen-presenting cells (APCs) in nearby lymphoid organs.^9^ Chemotherapy used in the treatment of malignancies^10^^,^^11^ and conditioning regimens prior to HSCT damages IECs and disrupts the epithelial barrier.^12^^,^^13^^,^^14^ After HSCT, the barrier breach may cause local inflammation and activation of T cells supporting the development of GVHD.^15^^,^^16^ The type and intensity of the conditioning regimen and regimen-induced toxicity correlate with the development and severity of GVHD, and related outcomes in patients.^17^^,^^18^^,^^19^^,^^20^

There is evidence that the intestinal epithelium and T cells closely interact under both homeostatic and pathogenic conditions, modulating T cell recruitment, differentiation, and function.^21^^,^^22^^,^^23^^,^^24^^,^^25^^,^^26^^,^^27^^,^^28^^,^^29^ T cells exhibit dynamic behavior within the IEC compartment which adapts to intraluminal epithelial cell exposure to pathogens.^30^^,^^31^ Intestine-derived IL-18 modulates inflammation by suppressing Th17 cells and stimulating T regulatory (Treg) cell differentiation.^26^^,^^27^ In acute GVHD, gut-directed α4β7-expressing T cells are preferentially recruited to intestinal crypts due to clustering of MAdCAM-1 expression on the endothelium of capillaries in the lower small intestinal crypt region making ISCs prone to damage.^29^^,^^32^ IECs have also been shown to play an essential role as APCs in murine gut GVHD^23^ determining local activation of CD4^+^ T cells through major histocompatibility class II (MHC-II)-antigen presentation.

Here, we have investigated the direct effects of intestinal epithelial damage caused by chemotherapy exposure on T cell behavior using a human intestinal organoid-based damage model. Intestinal organoids are 3D epithelial cultures that self-organize from isolated intestinal crypts when supplied with essential growth factors and have the potential to differentiate into all IEC types.^33^^,^^34^ We and others have demonstrated organoids to be a relevant *ex vivo* proxy for studying *in vivo* intestinal epithelial-immune cell interactions.^32^^,^^35^^,^^36^^,^^37^^,^^38^ In this study, we show that chemotherapy-induced epithelial injury transcriptionally reprograms IECs directly promoting T cell migration, proliferation, and activation. In addition, galectin-9 (Gal-9), a β-galactoside-binding lectin released by the damaged epithelium, was found to play a role in this IEC-mediated modulation of T cell behavior. Taken together, this study highlights the potential contribution of chemotherapy-induced intestinal epithelial damage and Gal-9 in particular to directly promoting T cell trafficking and increasing the magnitude of T cell activation upon stimulation. This could provide novel therapeutic targeting strategies to reduce damage-induced inflammatory responses.

## Results

### Modeling chemotherapy-induced damage in human small intestinal epithelial organoids

**Figure 1:**
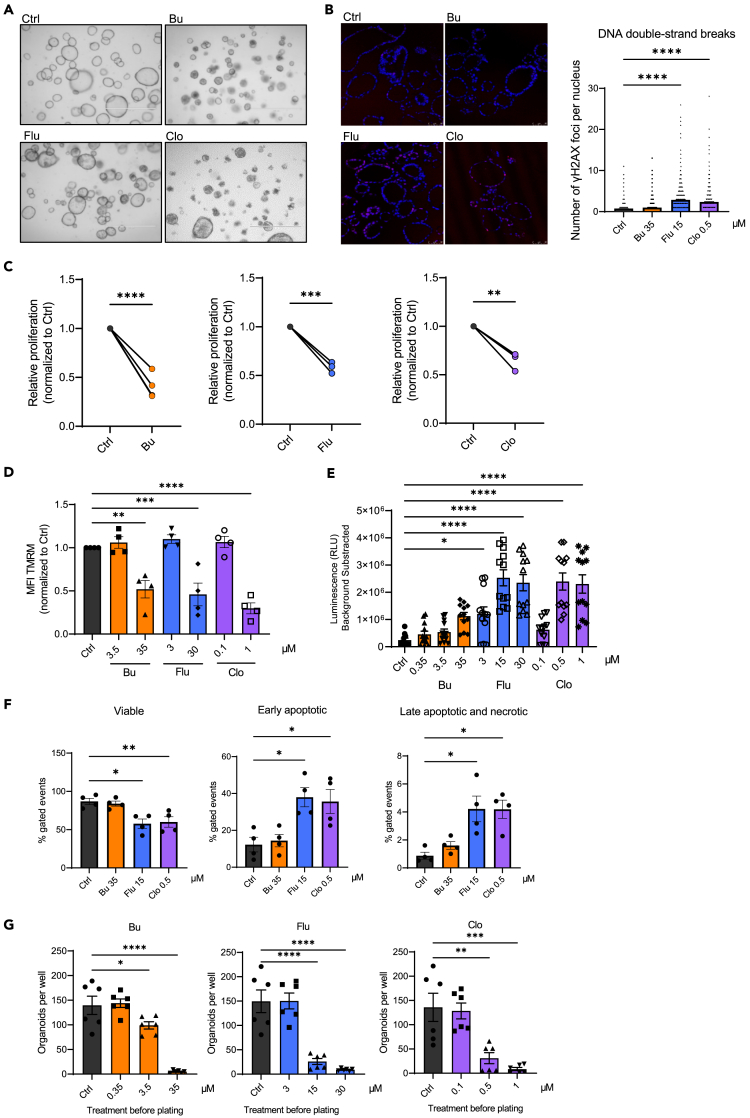
Modeling chemotherapy-induced damage in human small intestinal epithelial organoids (A) Representative EVOS images of organoids treated for 96 h with busulfan (Bu, 35 μM), fludarabine (Flu, 15 μM) or clofarabine (Clo, 1 μM), scale bar = 1000 μm. (B) Representative confocal images of organoids stained with DAPI (blue) and phosphorylated (γ) histone (H)2AX (γH2AX) complexes (red/AF647) 24 h after chemotherapy-treatment, at 40X, scale bar = 50 μm (left panel), quantification of γH2AX foci per nucleus (right panel) (*n* = 2 donors, >700 nuclei analyzed per condition), mean with SEM, ANOVA. (C) Relative proliferation of chemotherapy-treated organoids, quantified as CellTrace Violet MFI ratio by FACS after 72 h of Bu 35 μM (*n* = 4 donors), Flu 7.5 μM (*n* = 2 donors), or Clo 0.25 μM (*n* = 2 donors) treatment, Paired *t* test. (D) Normalized MFI of TMRM staining for functional mitochondria after 48 h of indicated chemotherapy treatment, measured by FACS, *n* = 2 donors, mean with SEM, ANOVA. (E) Levels of cleaved caspase 3/7 as measured by CaspaseGlo assay after 48 h of indicated chemotherapy treatment, *n* = 4 donors, mean with SEM, ANOVA. (F) Annexin-V and propidium iodide (PI) staining for early apoptotic (Annexin-V^+^) and late apoptotic or necrotic cells (Annexin-V^+^PI^+^) after 48 h of indicated chemotherapy treatment (right panels), measured by FACS. Representative FACS, *n* = 2 donors, mean with SEM, ANOVA. (G) Reconstitution of single organoid cells into organoids after treatment with indicated chemotherapeutics for 48 h, *n* = 2 donors, mean with SEM, ANOVA. Significance is indicated as *p* ≤ 0.05 (∗), *p* ≤ 0.01 (∗∗), or *p* ≤ 0.001 (∗∗∗) or *p* < 0.0001 (∗∗∗∗).

To study the *direct* effects of chemotherapy-induced epithelial damage on T cell behavior, we developed a damage model by exposing human intestinal epithelial organoids to three chemotherapeutics that are frequently used in HSCT conditioning regimens: busulfan (Bu), fludarabine (Flu), and clofarabine (Clo).^39^^,^^40^^,^^41^^,^^42^^,^^43^^,^^44^^,^^45^^,^^46^ Busulfan is an alkylating agent that crosslinks DNA strands, inhibiting DNA replication,^47^ while fludarabine and clofarabine are both nucleoside analogs (NA), which are incorporated into the DNA during normal synthesis and thereby stall replication.^48^ We evaluated chemotherapy concentrations that are used within the range of preclinical studies conducted in leukemia cell lines that eventually formed the basis for their clinical application.^40^^,^^49^ Four-day chemotherapeutic exposure resulted in visible morphological changes, with smaller organoid size, a condensed/folded phenotype and shedding of dead cells and cell debris both inward into the organoid lumen and outward into the organoid surroundings (Figure 1A). Sites of stalled DNA replication and DNA damage can be identified by nuclear foci of phosphorylated (γ) histone (H)2AX complexes.^50^ Indeed, exposure to all three chemotherapeutics caused a significant increase in γH2AX complexes per nucleus in treated organoids (Figures 1B and S1A). In treated organoids the induced replicative stress correlated with a reduction in proliferation compared to untreated organoids (Figure 1C). A dose-dependent decrease in the mitochondrial membrane potential was also observed (Figure 1D) indicative of increased oxidative stress and apoptosis. Indeed, chemotherapy-exposure induced apoptosis, as measured by a dose-dependent increase of caspase 3/7 activity (Figure 1E) and an increased percentage of Annexin-V-positive cells (Figure 1F). Functionally, the number of organoids generated from single cells was reduced, showing that the chemotherapy-treatment impaired their ability to regenerate (Figure 1G). Taken together, the tissue-damaging effects of chemotherapeutic-exposure on human intestinal epithelia can be modeled *ex vivo* using organoids.

### Chemotherapy treatment modulates intestinal transcriptional programs

**Figure 2:**
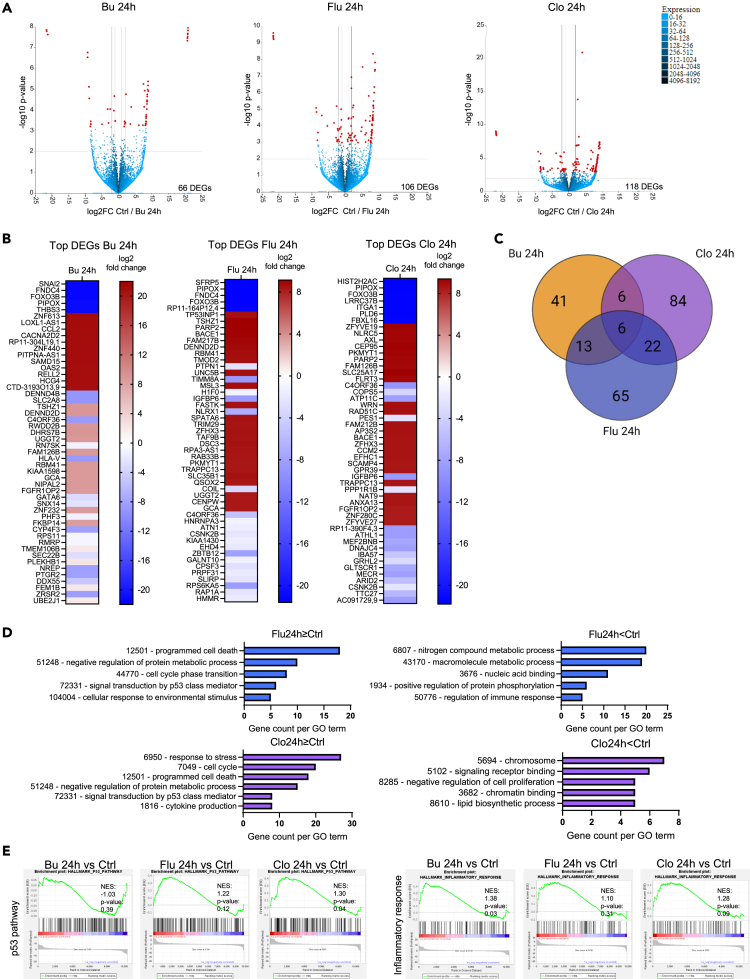
Chemotherapy conditioning specifically reprograms the intestinal epithelial transcriptome (A–E) Human intestinal epithelial organoids (*n* = 3 donors) were treated with busulfan (Bu, 3.5 μM), fludarabine (Flu, 15 μM), clofarabine (Clo, 0.5 μM) for 24 h. RNA was isolated and subjected to bulk RNA-sequencing, after which bioinformatics analyses was performed. (A) Volcano plots indicating differentially expressed (padj<0.1) genes of 24 h Bu- (left), Flu- (middle) and Clo- (right) treated organoids versus control in red. (B) Heatmaps of top most different differentially expressed genes in 24 h Bu- (left), Flu- (middle) and Clo- (right) treated organoids versus control (DESeq2, padj<0.1). (C) Venn-diagram showing numbers of overlapping and distinct DE genes between different chemotherapeutics (DESeq2, padj<0.1). Illustration made with BioRender.com. (D) Gene Ontology (GO) term analysis of Biological Processes in genes upregulated (LFC≥0) or downregulated (LFC<0) by Flu- (top) and Clo- (bottom) treatment of organoids (padj<0.1). (E) Gene Set Enrichment Analysis (GSEA) on all genes in each indicated treatment-condition.

To evaluate transcriptional responses to chemotherapeutic exposure, bulk RNA sequencing (RNA-seq) of three independent organoid donors was performed. Treatment of organoids with Bu, Flu, or Clo for 24 h resulted in relatively subtle transcriptional changes with 66, 106, and 118 differentially expressed genes (DEGs) respectively (Figure 2A; Table S1). A concise list of the top 50 DEGs (25 most upregulated and 25 most downregulated genes) identified for each individual treatment is shown in Figure 2B. While there are considerable differences between chemotherapeutics, all share certain similarities, in particular the two nucleoside analogs Flu and Clo, reflecting their common mechanism of action (Figures 2C and S1B). Flu- and Clo-treated organoids share the following DEGs: *BACE1, BAX, CSNK2B, DDB2, GADD45A, GDF15, H1F0, IGFBP6, KIAA1430, KIAA1432, KIF3C, LAPTM4B, MDM2, PARP2, PKMYT1, RRM2B, TP53INP1, TRAPPC13, TXNIP, TYMS, UBR7,* and *ZFHX3*. Bu-, Flu-, and Clo-treated organoids share the following DEGs: *C4ORF36, DENND2D, FAM126B, FOXO3B, PIPOX, SLC40A1*. The differential expression of several of these genes was evaluated by quantitative reverse transcription PCR (qRT-PCR) (Figure S2). Gene Ontology (GO) term analysis of upregulated genes indicated a similar over-representation of upregulated gene sets in the nucleoside analog Flu- and Clo-treated organoids, including (mitotic) cell cycle processes, metabolic processes, and programmed cell death (Figure 2D; a complete list of GO gene sets is provided in Table S1). Reflecting their mechanism of action, over-representation of genes related to DNA damage and repair as well as the p53 pathway was predominant in Flu- and Clo-treated organoids. Differences between the chemotherapy types, nucleoside analog or alkylating agent, were also observed by Gene Set Enrichment Analysis (GSEA) of chemotherapy-treated organoids (Figure 2E). For example, a negative association between Bu-treatment and the p53 pathway was observed, in contrast to Flu- and Clo-treatment. However, a transcriptional change associated with inflammatory response was observed for all treatments. In summary, chemotherapeutics can evoke distinct transcriptional responses in the intestinal epithelium with nucleoside analogs Flu and Clo being more similar when compared with the alkylating agent Bu.

### Chemotherapy-damaged organoids directly promote T cell responses

**Figure 3:**
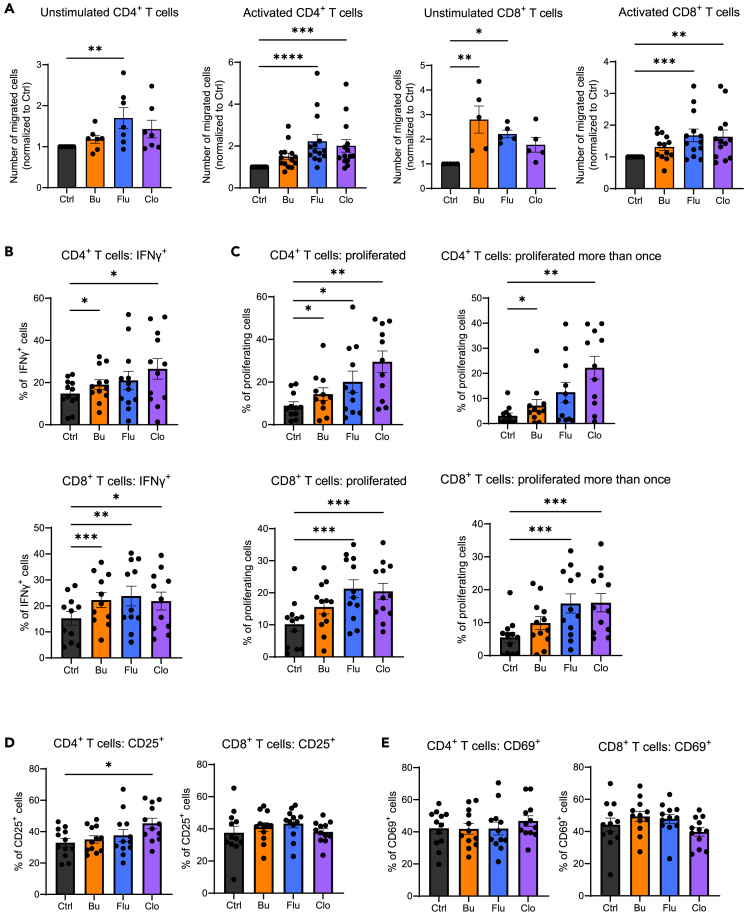
Chemotherapy-induced damage promotes T cell migration and activation (A) Normalized number of unstimulated and pre-activated CD4^+^ and CD8^+^ T cells that have migrated overnight from a 3 μm-pore sized insert (upper compartment) to the lower compartment containing organoids that were treated with busulfan (Bu, 35 μM), fludarabine (Flu, 15 μM), clofarabine (Clo, 0.5 μM) for 48 h, and then refreshed for 24 h, counted by FACS. *n* ≥ 5 T cell donors with 1 organoid donor, each data point indicates a T cell donor, mean with SEM, ANOVA. (B–E) (Membrane) activation marker expression and proliferation of CD4^+^ and CD8^+^ T cells as measured by CellTrace Violet-dilution after 4-day co-culture with organoids that were previously treated with Bu (35 μM), Flu (15 μM), Clo (0.5 μM) for 24 h before the start of the assay. Organoids were washed and disrupted mechanically before replating in co-culture with T cells. *n* ≥ 11 T cell donors with 1 organoid donor, each data point indicates a T cell donor, mean with SEM, ANOVA. Significance is indicated as *p* ≤ 0.05 (∗), *p* ≤ 0.01 (∗∗), or *p* ≤ 0.001 (∗∗∗) or *p* < 0.0001 (∗∗∗∗).

To evaluate whether chemotherapy-damaged organoids can directly influence T cell responses, we first evaluated migration (Figure S3A). Intermediate chemotherapeutic concentrations were chosen to model epithelial damage. To quantify T cell migration, we made use of a transwell system with 3μm-pored inserts. Organoids were treated with chemotherapeutics for 48 h and subsequently cultured in drug-free medium for an additional 24 h. Either unstimulated or polyclonally pre-activated CellTrace Violet (CTV)-stained human peripheral blood CD4^+^ and CD8^+^ T cells were subsequently added to the transwell insert (Figure S3B). After 24 h, the number of T cells that had migrated to the lower compartment was evaluated. Both unstimulated and pre-activated CD4^+^ and CD8^+^ T cells demonstrated significantly increased migration toward chemotherapy-treated organoids (Figure 3A).

Subsequently, the effect of chemotherapy-damaged epithelium on the polyclonal activation of T cells was evaluated (Figure S3C). CD4^+^ and CD8^+^ T cells were isolated, stained with CTV, and activated by incubation with plate-bound anti-CD3 and soluble anti-CD28, in the presence of untreated or chemotherapy-treated organoids (representative images of organoids after treatment and before washing are shown in Figure S3D). This co-culture system allows measurement of the direct effects of epithelial damage on T cell activation, as T cells received activation stimuli in the presence of damaged organoids. More specifically, polyclonal activation allows us to evaluate the co-stimulatory role of intestinal epithelial chemotherapy-induced damage regardless of antigen-specific reactions. Proliferation and activation markers were assessed by flow cytometry (FC) after four days of co-culture. Co-culture with chemotherapy-treated epithelial organoids led to both increased IFNγ-production (Figure 3B) and proliferation (Figure 3C). However, membrane expression levels of activation markers CD25 and CD69 (Figures 3D and 3E) were not significantly changed between conditions, with the exception of CD4^+^ T cells co-cultured with Clo-treated organoids which expressed higher CD25. In conclusion, Bu-, Flu-, and Clo-damaged intestinal epithelium promoted T cell migration, and potentiated T cell activation, supporting T cell expansion and IFNγ production.

### Intestinal organoid-derived galectin-9 promotes T cell activation and migration

**Figure 4:**
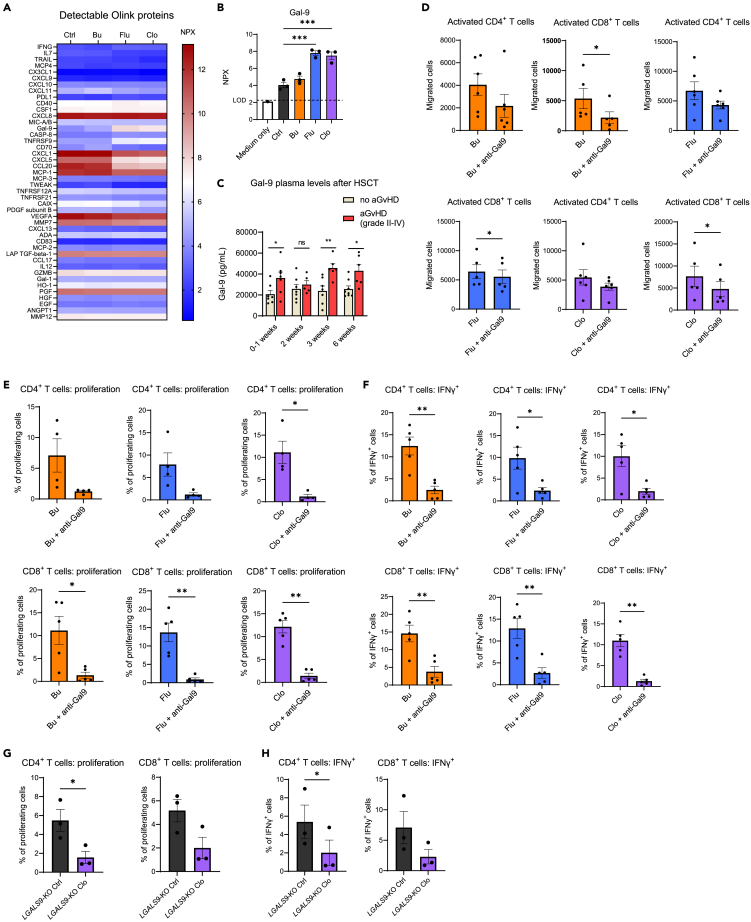
Galectin-9 released by chemotherapy-damaged organoids modulates T cell activation, migration, and proliferation (A and B) Analysis of proteins present in conditioned media of organoids treated with chemotherapeutics busulfan (Bu), fludarabine (Flu), and clofarabine (Clo) as in the *ex vivo* T cell migration assay (Olink Proteomics) (*n* = 3 donors). Each data point indicates the levels of galectin-9 (Gal-9) in the conditioned medium from each organoid condition (log scale) as normalized protein expression (NPX) units, mean with SEM, ANOVA. LOD: level of detection. (C) Gal-9 levels in plasma of HSCT patients with and without acute grade II-IV (gut) GVHD as measured by Luminex. *n* ≥ 5 patients per condition, mean with SEM, multiple *t* tests. (D) Migration of activated CD4^+^ and CD8^+^ T cells toward treated organoids in the presence of anti-Gal-9 blocking monoclonal antibody (mAb). *n* ≥ 5 T cell donors with 1 organoid donor, each data point indicates a T cell donor, mean with SEM, paired *t* test. (E) CD4^+^ and CD8^+^ T cell proliferation after co-culture with treated organoids in the presence of anti-Gal-9 mAb. *n* ≥ 4 T cell donors with 1 organoid donor, each data point indicates a T cell donor, mean with SEM, paired *t* test. (F) CD4^+^ and CD8^+^ T cell intracellular IFNγ after co-culture with treated organoids in the presence of anti-Gal-9 mAb. *n* ≥ 5 T cell donors with 1 organoid donor, each data point indicates a T cell donor, mean with SEM, paired *t* test. (G) CD4^+^ and CD8^+^ T cell proliferation after co-culture with Clo-treated *LGALS9*-knock-out (KO) organoids. *N* = 3 T cell donors with 1 organoid donor, each data point indicates a T cell donor, mean with SEM, paired *t* test. (H) CD4^+^ and CD8^+^ T cell intracellular IFNγ after co-culture with Clo-treated *LGALS9*-KO organoids. *N* = 3 T cell donors with 1 organoid donor, each data point indicates a T cell donor, mean with SEM, paired *t* test. Significance is indicated as *p* ≤ 0.05 (∗), *p* ≤ 0.01 (∗∗), or *p* ≤ 0.001 (∗∗∗) or *p* < 0.0001 (∗∗∗∗).

To gain mechanistic insight into how epithelial damage can increase the migration and proliferation of T cells, we utilized the Olink proteomics platform. Here, we evaluated conditioned medium (CM) from both chemotherapy-damaged and untreated intestinal organoids.^51^ 44 proteins were above level of detection (LOD), including chemokines such as C-X-C motif chemokine ligand 9 (CXCL9), CXCL10, and CXCL11; cytokines such as IFNγ and IL-8; immune checkpoint molecules such as Programmed death-ligand 1 (PD-L1), and growth factors including EGF among others (Figure 4A). Galectin-9 (Gal-9), a β-galactoside-binding lectin, was detected in the CM from organoids, and significantly increased in the CM from Flu- and Clo-treated organoids (Figure 4B).

To evaluate whether Gal-9 levels following conditioning are also increased in a clinical setting, we measured Gal-9 levels in stored plasma samples of 17 pediatric transplant patients aged 12–18 at multiple early time points after HSCT. All patients received a conditioning regimen with Bu and Flu, and, in addition, some patients Clo, before undergoing transplantation with a cord blood graft. Seven patients developed acute GVHD grade II-IV with gastrointestinal involvement. Median time to GVHD was 24 days (range 15–45) and all patients had a follow-up of more than 100 days. Gal-9 levels were measurable in the plasma of all patients (Figure 4C) and were increased at the day of, or early after, transplant in patients that later developed grade II-IV acute GVHD. Furthermore, Gal-9 levels remained elevated at several time points after HSCT in grade 2–4 GVHD patients. While correlative, this observation supports a role for Gal-9 as an intestinal damage biomarker.

To determine whether Gal-9 may play a role in mediating the effects of chemotherapy-damaged organoids on T cell responses, an anti-Gal-9 blocking monoclonal antibody was utilized. Anti-Gal-9 added to the lower compartment significantly inhibited CD8^+^ T cell migration and showed a trend toward decreased migration of CD4^+^ T cells (Figure 4D). Furthermore, the presence of anti-Gal-9 abrogated both increased proliferation (Figure 4E) and IFNγ levels (Figure 4F) observed in the presence of Bu-, Flu-, and Clo-treated organoids. In contrast, T cells preserved the ability to express activation markers CD69 and, to a certain extent, CD25 (Figure S4A). To further confirm and validate these observations, we generated an organoid knock-out (KO) line for *LGALS9*, the gene encoding Gal-9. We confirmed the absence of expression of Gal-9 in this line after several weeks from electroporation, demonstrating the genetic ablation (Figure S4B). *LGALS9*-KO organoids were exposed to Clo, chosen because it was the chemotherapeutic most consistently providing a strong immunostimulatory effect on T cell activation, and then subsequently co-cultured with CD4^+^ and CD8^+^ T cells under polyclonal activation conditions. We observed a trend of decreased activation and proliferation of cells (Figures 4G, 4H, and S4C) when exposed to Clo-treated organoids compared to untreated control organoids when organoid-derived Gal-9 was absent. Taken together, these data support a pathogenic role for Gal-9 by increasing migration and activation of T cells at the site of the chemotherapy-damaged intestinal epithelium.

## Discussion

Immune activation after IEC injury is a well-known phenomenon, but how such damage can directly affect T cell function remains unclear. Here we have developed an *ex vivo* intestinal organoid model to mimic chemotherapy-induced damage allowing us to study the direct effect of epithelial injury on T cell homeostasis. In addition to developing our understanding of the biology of T cell responses during sterile inflammation, the model has the potential to serve as a platform for the discovery of new targets and testing of therapeutics in multiple intestinal disease settings, as well as in the tailored development of autologous immunotherapy and chimeric antigen receptor (CAR) T cell therapy for solid tumors in sequence with chemo- or radiotherapy.^52^^,^^53^^,^^54^^,^^55^

T cell activation and associated tissue damage is known to occur also independent of antigen presentation by IECs.^56^ We have previously shown that dysregulated T cell activation and IFNγ production are potent inducers of a JAK/STAT-mediated intestinal (stem cell) damage.^32^ Our current data demonstrate that prior chemotherapy-induced damage results in the release of Gal-9 from intestinal epithelia. Gal-9 can directly modulate the level of T cell responses with higher production of IFNγ and increased proliferation in the context of polyclonal activation, suggesting a co-stimulatory or priming effect regardless of antigen-specific reactions. In this way, chemotherapy-induced antigen-independent signals influence adaptive immunity in absence of other cellular mediators. Gal-9 is therefore a potential biomarker of intestinal damage and a possible therapeutic target.

This is the first study comparing epithelial damage response to different clinically relevant chemotherapeutics. We observed that exposure of intestinal epithelial organoids to chemotherapeutics resulted in chemotherapy-distinct transcriptional responses. The major chemotherapeutic mechanism of action of Flu and Clo is chain termination when incorporated in place of natural purine nucleosides, with stalling of the cells in S-phase resulting in induction of apoptosis. Bu on the other hand is an alkylating agent that crosslinks guanine bases in the DNA, therefore, making it impossible for DNA strands to unfold, thereby also resulting in apoptosis. Although we cannot completely exclude the contribution of the chemotherapeutic concentrations utilized, we observed a similar gene expression pattern and related T cell responses between Flu and Clo, the purine NAs we included in our study. While it is currently not possible to reduce chemotherapy regimens, it is important to understand the differential consequences of exposure to each individual chemotherapeutic, and a further argument for pursuing the development of antibody-based conditioning for the future.^57^

We found that epithelial damage caused by chemotherapy increases the migration of both unstimulated and polyclonally pre-activated T cells toward the site of injury. The migration assay studies the cells in two distinct compartments, suggesting a soluble molecule gradient to be responsible for this. Interestingly, in our setting, increased migration occurred in the absence of endothelium, or any previously known intestinal-epithelial-derived chemokine. We did not observe an increase in the levels of the chemokines CXCL9, CXCL10, and CXCL11 as measured by Olink proteomic analysis. However, we observed decreased T cell migration when blocking Gal-9 during the migration assays, suggesting a possible role for Gal-9 in modulating T cell trafficking to the damaged epithelium.

Besides the capacity of chemotherapy-induced epithelial damage to increase T cell migration, we also demonstrate a direct influence on the proliferation and activation of T cells. Besides peripheral and local hematopoietic APC-mediated activation, we have shown for the first time that damaged epithelium can directly additionally stimulate tissue-recruited activated T cells, which likely further propagates intestinal damage through local IFNγ production. While the precise mechanism by which this occurs remains to be fully elucidated, we have identified Gal-9 as novel driver. Gal-9 is a β-galactoside-binding lectin with immunomodulatory properties widely expressed by a variety of tissues.^58^^,^^59^^,^^60^ The carbohydrate domains of Gal-9 can bind β-galactosides, such as lactose, found on O-glycans and N-glycans of glycosylated proteins or lipids.^61^ Gal-9 expression can be nuclear as well as cytoplasmic, or extracellular where it is membrane-bound or in the extracellular matrix. Gal-9 has many different receptors, among which TIM-3, DR3, 4-1BB, CD44, PD-1 and Protein Disulfide Isomerase are expressed on T cells.^62^^,^^63^^,^^64^^,^^65^^,^^66^^,^^67^ Interestingly, the expression of soluble TNFRSF9 (CD137 or 4-1BB), one of the receptors for Gal-9, was also significantly upregulated in the conditioned media of chemotherapy-treated organoids (Figure S4D). The reported effects of Gal-9 on T cells *in vitro*, *in vivo,* and deduced from human biomarker studies are pleiotropic and range from immunosuppressive to pro-inflammatory. The complexity of reported effects may be related to the Gal-9 doses utilized, species differences, as well as the cellular or tissue context. Gal-9 at low doses has been shown to increase T cell expansion and activation *ex vivo*, inducing a Th1 phenotype with IFNγ production.^68^ This Th1-type response by Gal-9 has been linked to a pathway mimicking antigen-specific activation of the TCR resulting in cytosolic calcium mobilization.^69^^,^^70^^,^^71^ While the precise mechanism behind these effects is still not completely clear, the binding of Gal-9 to TIM-3 on conventional T cells has been reported to induce apoptosis.^62^ However, TIM-3-induced apoptosis by Gal-9 has only been observed under supraphysiological concentrations of Gal-9, and the relevance of this for human T cells is unclear. These effects are also likely to be immune context-dependent since CD4^+^ T cells from rheumatoid arthritis patients also do not undergo apoptosis when exposed to increasing concentrations of Gal-9, compared to cells from healthy controls.^72^ In addition, Gal-9 has the property of oligomerizing and forming lattices, which might contribute to the resulting effect in a complex way by modulating other receptors local concentration.^73^ For example, co-expression of PD-1 and TIM-3 has been shown to prevent TIM-3-mediated apoptosis triggered by Gal-9 through the formation of lattices comprising PD-1, TIM-3, and Gal-9.^67^

Damage to the gastrointestinal tract plays a major role in the morbidity and mortality associated with GVHD. Our data show increased plasma levels of Gal-9 even early after conditioning which persist in patients that develop GVHD after HSCT. Others have shown increased serum Gal-9 later, at onset of GVHD, suggesting it to be a biomarker of the disease, which may or may not be directly related to its pathophysiology.^74^ Gal-9 levels have also been associated with intestinal inflammation and correlate with disease severity in IBD.^75^ In murine GVHD models, Gal-9 has been shown to have divergent effects, ranging from being protective,^76^ to being detrimental in the absence of Treg cells.^77^ Gal-9 has also recently received attention in the context of cancer immunotherapy^67^^,^^78^ where anti-Gal-9 blocking antibodies are being developed as checkpoint inhibitors to induce the anti-tumor immune response. Ongoing clinical trials (NCT04666688) will provide important information regarding the safety and effectiveness of Gal-9 blockade, potentially helping the translation of Gal-9 targeting intervention for GVHD patients.^79^ However, given the pleiotropic and diverse effects reported, strict control of Gal-9 perturbation must be in place in respect to timing, location and cell types involved.

In conclusion, we have modeled chemotherapy-induced damage to the human intestinal epithelium and studied its direct effects on T cell homeostasis. T cells demonstrated increased migration to chemotherapy-treated organoids, proliferated more and expressed higher levels of IFNγ. We propose damaged-organoid-derived Gal-9 as a damage-associated molecule responsible for these effects. While outside of the scope of this study, we suggest that Gal-9 may be a potential biomarker for intestinal damage and inflammation. In addition, these findings support exploring Gal-9-signaling perturbation as a damage-dampening, preventive therapy in the context of immune-mediated diseases.

### Limitations of the study

Besides chemotherapy, radiotherapy is also used in conditioning regimens prior to HSCT under specific transplant indications (specifically acute lymphatic leukemia and lymphoma).^46^ Others have shown that irradiation causes damage to organoids and reduces their proliferation in a similar fashion as to our findings.^80^ While outside the scope of our study, our model system could also be adapted to understand the crosstalk between irradiated IECs and T cells. In addition, we identified organoid-derived Gal-9 as factor modulating T cell behavior. However, we did not determine which Gal-9 receptors are responsible for the effects on T cells reported here. Further research is required to clarify the mechanistic properties of Gal-9 in the presented model system, considering the different known Gal-9 receptors. This understanding could be exploited to prevent allo-T cell responses in HSCT settings and should be addressed in the future.

## STAR★Methods

### Key resources table

**Table:** 

REAGENT or RESOURCE	SOURCE	IDENTIFIER
**Antibodies**		

Functional grade anti-human CD3 (OKT3)	eBioscience	Cat: 16-0037-85; RRID: AB_468855
Functional grade anti-human CD28 (CD28.2)	eBioscience	Cat: 16-0289-85; RRID: AB_468927
anti-human Galectin-9 (9M1-3)	Biolegend	Cat: 348902; RRID: AB_10612753
rabbit anti-phospho-histone H2A.X (Ser139) (20E3)	Cell Signaling	Cat: 9718; RRID: AB_2118009
goat anti-rabbit Alexa 647	Thermo Fisher	Cat: a21244; RRID: AB_2535812
PE anti-human CD3 Antibody (UCHT1)	BioLegend	Cat: 300408; RRID: AB_314062
FITC anti-human CD4 Antibody (RPA-T4)	BioLegend	Cat: 300506; RRID: AB_314074
FITC anti-human CD8 Antibody (SK1)	BioLegend	Cat: 344704; RRID: AB_1877178
APC anti-human CD25 Antibody (BC96)	BioLegend	Cat: 302610; RRID: AB_314280
Brilliant Violet 605 anti-human CD69 (FN50)	BioLegend	Cat: 310937; RRID: AB_2562306
PE-Cy™7 anti-Human IFN-γ (4S.B3)	BD Biosciences	Cat: 557844; RRID: AB_396894
anti-Galectin-9 (EPR22214)	Abcam	Cat: ab227046; RRID: AB_2883983
anti-β-Actin (C4)	Santa Cruz	Cat: sc-47778; RRID: AB_626632
Swine Anti-Rabbit Immunoglobulins/HRP	Agilent	Cat: P0217; RRID: AB_2728719
Rabbit Anti-Mouse Immunoglobulins/HRP	Agilent	Cat: P0260; RRID: AB_2636929

**Biological samples**		

Peripheral blood mononuclear cells	UMC Utrecht; Sanquin bloodbank	N/A
Plasma samples from HSCT patients	UMC Utrecht biobank	N/A

**Chemicals, peptides, and recombinant proteins**		

Ficoll-Paque	GE Healthcare	Cat: 17-1440-03
Matrigel	Corning	Cat: 356231
Advanced DMEM/F-12	Gibco	Cat. 12634010
RPMI 1640 Medium, GlutaMAX™ Supplement	Gibco	Cat. 61870010
Recombinant murine EGF	Prepotech	Cat: 315-09;
Molecular Biology Grade Agarose	Eurogentec	Cat: EP-0010-05; CAS: 9012-36-6
HEPES	Gibco	Cat: 15630-080
TrypLE Express	Gibco	Cat: 12604-021
Glutamax	Gibco	Cat: 35050-038
Nicotinamide	Sigma	Cat: N0636-500G; CAS: 98-92-0
N-acetyl cysteine	Sigma	Cat: A9165-100G; CAS: 616-91-1
B27	Gibco	Cat: 17504-044
TGF-β inhibitor A83-01	Tocris	Cat: 2939/10; CAS: 909910-43-6
p38 inhibitor SB202190	Sigma	Cat: S7067-25 mg; CAS: 152121-30-7
Rho-kinase/ROCK inhibitor Y-27632	Abcam	Cat: ab120129-10; CAS: 129830-38-2
Busulfan	Busilvex	Cat: N/A; CAS: 55-98-1
Busulfan	TEVA	Cat: N/A; CAS: 55-98-1
Fludarabinephosphate	Aerobindo	Cat: N/A; CAS: 21679-14-1
Clofarabine	Evoltra	Cat: N/A; CAS: 123318-82-1
Clofarabine	Mylan	Cat: N/A; CAS: 123318-82-1
Primocin	Invivogen	Cat: ant-pm-2
Penicillin-Streptomycin	Gibco	Cat: 15070063; CAS: 3810-74-0
GolgiStop™ Protein Transport Inhibitor (containing Monensin)	BD Biosciences	Cat: 554724; CAS Monensin: 22373-78-0
Basement Membrane Matrix (BME)	Cultrex	Cat: 3533-005-02
DAPI	Sigma Aldrich	Cat: F6057
Paraffin (Leica Paraplast)	Leica Biosystems	Cat: 39602012
Ethanol 100%	Boom	Cat: 84050065.5000; CAS: 64-17-5
Ethanol 96%	Boom	Cat: 84045206.5000; CAS: 64-17-5
Ethanol 70%	Boom	Cat: 84010059.5000; CAS: 64-17-5
Sodium citrate buffer	Merck	Cat: F2050643 941
Zombie NIR	BioLegend	Cat: 423106
MitoTracker Green FM	Thermo Fisher	Cat: M7514; CAS: 201860-17-5
Tetramethylrhodamine methyl ester perchlorate (TMRM)	Thermo Fisher	Cat: T5428; CAS: 115532-50-8
UltraPure™ Ethylenediaminetetraacetic Acid, Disodium Salt, Dihydrate (Na_2_EDTA·2H_2_O)	Invitrogen	Cat. 15576-028; CAS: 6381-92-6
Natriumchloride (NaCl)	Boom	Cat: 76028327.5000; CAS: 7647-14-5
Kaliumchloride (KCl)	Boom	Cat: 76050944.1000; CAS: 7447-40-7
Natriumhydroxide (NaOH)	Boom	Cat. 76028323.1000; CAS: 1310-73-2
Hydrogenchloride (HCl)	Boom	Cat: 76021889.2500; CAS: 7647-01-0
Fetal Bovine Serum (FBS) South America origin	Biowest	Cat. S1810-500
Tween 20	Sigma-Aldrich	Cat. 8221841000; CAS: 9005-64-5
30% Acrylamide/Bis Solution 37.5:1	BioRad	Cat: #1610158; CAS (Acrylamide): 79-06-1; CAS (Methylene diacrylamide): 110-26-9

**Critical commercial assays**		

CD8^+^ Dynabeads isolation kit	Thermo Fisher	Cat: 11333D
MagniSort human CD4^+^ T cell enrichment kit	Thermo Fisher	Cat: 8804-6811-74
CellTrace Violet	Invitrogen	Cat: C34557
Caspase-Glo 3/7 Reagent	Promega	Cat: G8091
Dead Cell Apoptosis Kit with Annexin V-FITC and propidium iodide	Thermo Fisher	Cat: V13242
Intracellular Fixation & Permeabilization Buffer Set	eBioscience	Cat: 88-8824-00
Halt™ Protease Inhibitor Cocktail (100X)	Thermo Fisher	Cat: 1861278
SuperSignal West Femto Extended Duration Substrate	Thermo Fisher	Cat: 34096
SuperSignal West Dura Extended Duration Substrate	Thermo Fisher	Cat: 34076
iScript cDNA Synthesis Kit	BioRad	Cat: 1708890
TaqMan Fast Advanced Master Mix for qPCR	Applied Biosystems	Cat: 4444556
TaqMan probe *GADD45A*	Thermo Fisher	ID: Hs00169255_m1
TaqMan probe *HP1BP3*	Thermo Fisher	ID: Hs00212448_m1
TaqMan probe *LGALS9*	Thermo Fisher	ID: Hs00371321_m1
TaqMan probe *MDM2*	Thermo Fisher	ID: Hs00540450_s1
TaqMan probe *PARP2*	Thermo Fisher	ID: Hs00193931_m1

**Deposited data**		

RNA-seq chemotherapy-treated organoids	This manuscript	https://ega-archive.org/; EGA: EGAS00001007550

**Experimental models: Cell lines**		

Small intestinal organoids	UMC Utrecht biobank	N/A

**Oligonucleotides**		

gRNA *LGALS9* TTTGCTGTGAACTTTCAGAC (PAM: TGG)	IDT	Design ID: Hs.Cas9.LGALS9.1.AC

**Software and algorithms**		

Fiji (ImageJ 1.53q)	Schindelin et al.^81^	https://fiji.sc/
ImageJ script used to analyze γH2AX-foci	Segeren et al.^82^	N/A
CytExpert 2.4	Beckman Coulter	https://www.beckman.pt/flow-cytometry/research-flow-cytometers/cytoflex/software
FlowJo 10.6.2	BD; Becton, Dickinson and Company 2023	https://www.flowjo.com
R2: Genomics Analysis and Visualization Platform		https://r2.amc.nl
GSEA Software	Subramanian et al.^83^	https://www.gsea-msigdb.org/gsea/index.jsp
Prism 9	GraphPad	https://www.graphpad.com

**Other**		

Thincert cell culture insert for 24 well plates, tc, sterile, transparent membrane (pet), pore diameter: 3 μm	Greiner Bio-One	Cat. 662630

### Resource availability

#### Lead contact

Further information and requests should be directed to and will be fulfilled by the lead contact, Caroline A. Lindemans C.A.Lindemans@prinsesmaximacentrum.nl.

#### Materials availability

This study did not generate new unique reagents.

#### Data and code availability

• Data: All data reported in this paper will be shared by the lead contact upon request. The RNA-seq dataset analyzed in this manuscript is uploaded on EGA (EGA: EGAS00001007550).
• Code: This paper does not report original code.
• Any additional information required to reanalyze the data reported in this paper is available from the lead contact upon request.

### Experimental model and study participant details

T cells were obtained from peripheral blood of healthy donors. All individuals provided written informed consent as approved by the medical ethical review board of the UMC Utrecht (METC) (protocol METC 07–125/O). Alternatively, T cells were also obtained from buffy coats purchased from Sanquin. All individuals provided written informed consent (project NVT0597.01).

Biobanked small intestinal epithelial organoids were obtained from biopsies of patients originally tested for coealiac disease but declared healthy. All individuals provided written informed consent to participate in the study as approved by the medical ethical review board of the UMC Utrecht (METC) (protocol METC 10–402/K; TCBio 19–489).

Biobanked plasma samples from HSCT patients at specific time points related to their HSCT date were used for Luminex analysis. The data were collected after patients provided written informed consent (HSCT Biobank, local IRB approval 05–143 and 11-063k) in accordance with the Helsinki Declaration.

### Method details

#### T cell isolation and activation

T cells were isolated from peripheral blood of healthy donors in the UMC Utrecht as approved by the METC (protocol 07/125) or from buffy coats (Sanquin, NL). After Ficoll-Paque (GE Healthcare) gradient separation, CD8^+^ T cells were isolated from the peripheral blood mononuclear cells (PBMCs) in MACS buffer [phosphate buffered saline without calcium and magnesium [PBS0, made with milliQ, NaCl (8.0 g in 1L), KCl (0.2 h in 1L), pH adjusted to 7.25–7.3 with HCl], 2 mM EDTA (stock solution 0.5M, pH adjusted to 8.0 with NaOH), 2% heat-inactivated FBS] using the CD8^+^ Dynabeads isolation kit (Thermo Fisher) and BD IMag Cell Separation Magnet (BD Biosciences). CD4^+^ T cells were isolated from the CD8-depleted PBMC fraction using the MagniSort human CD4^+^ T cell enrichment kit (Thermo Fisher). T cell purity was checked by flow cytometry (routinely >80%, Figure S3B). T cells were activated using plate-bound functional grade anti-human CD3 (1.6 μg/mL in PBS0 overnight at 4°C or 2 h at 37°C, eBioscience) and soluble functional grade anti-human CD28 (1 μg/mL, eBioscience) for 3 or 4 days as indicated at a concentration of 1 × 10^6^ cells/mL in T cell medium (TCM) (RPMI Medium 1640+GlutaMAX, Gibco, with 100 U/mL pen-strep and 10% heat-inactivated FBS).

#### Intestinal organoid cultures

Healthy human small intestinal epithelial organoids were established and cultured as previously described.^34^ In short, organoids were generated from biopsies of individuals initially suspected of celiac disease, but declared free of pathology, and stored in a biobank. All individuals provided written informed consent to participate in the study as approved by the medical ethical review board of the UMC Utrecht (METC) (protocol METC 10–402/K; TCBio 19–489). Organoids (>passage 7) were passaged via single cell dissociation using 1× TrypLE Express (Gibco) and resuspended in Advanced DMEM/F12 (Gibco), 100 U/mL penicillin-streptomycin (Gibco), 10 mM HEPES (Gibco) and Glutamax (Gibco) (GF- medium), and 50–66% Matrigel (Corning). After plating and Matrigel polymerization, human small intestinal organoid expansion medium (hSI-EM) was added consisting of GF-, Wnt-3a conditioned-medium (CM) (50%), R-spondin-1 CM (20%), Noggin CM (10%), murine EGF (50 ng/mL, Peprotech), nicotinamide (10 mM, Sigma), N-acetyl cysteine (1.25 mM, Sigma), B27 (Gibco), TGF-β inhibitor A83-01 (500 nM, Tocris), p38 inhibitor SB202190 (10μM, Sigma), Rho-kinase/ROCK inhibitor Y-27632 (10μM, Abcam, for the first 2–3 days of culture), and Primocin (optional) (100 μg/mL, Invivogen). Medium was refreshed every 2–3 days. For indicated timepoints, treatment wells received different concentrations of Busulfan (Busilvex or TEVA), Fludarabinephosphate (Aerobindo) and Clofarabine (Evoltra or Mylan).

#### Migration transwell assay

Organoids were cultured in 24-well plates and treated for 48 h with indicated conditions. After treatment, the medium was refreshed with hSI-EM without p38 inhibitor (no SB) for 24 h. Simultaneously, isolated T cells were activated for 3 days or left unstimulated in TCM. At the start of the assay, T cells were stained with CellTrace Violet (CTV) (Invitrogen, 5 μM in PBS0 according to manufacturer’s protocol) and added to 3μm-pored transwell inserts (Greiner Bio-One) (4 × 10^5^ T cells in 200μL) that were placed in the wells with organoids. After overnight incubation, inserts were removed and the contents of each well was dissociated with TrypLE and reconstituted in 300μL. The number of CTV^+^ events per 150μL sample was counted using flow cytometry. For the Gal-9 blocking assays, 2 μg/mL anti-Gal-9 mAb (BioLegend) was added to the lower compartment prior to start of the assay.

#### T cell activation co-culture assay

For the evaluation of T cell activation and proliferation in the presence of chemotherapy treated organoids, organoids were cultured and treated with the indicated condition for 24 h. Subsequently, organoids were mechanically disrupted. A portion of each condition was used to dissociate into single cells to infer the cell number and normalize between different conditions. T cells were isolated and stained with CTV. Co-cultures were set up in a 96-well plate, with 2 × 10^5^ T cells and the equivalent of 1/6^th^ of untreated organoids per well, in 200 μL organoid medium without p38 inhibitor and supplemented with 10% BME (Cultrex). In activating conditions, wells had previously been coated with anti-CD3 and the medium was supplemented with anti-CD28. After 4 days of co-culture, plates were cooled at 4°C for 30 min to dissolve BME *in situ* and centrifuged at 500 g 5 min at 4°C. When performing intracellular staining, GolgiStop (containing Monensin, BD Biosciences) was added to the medium 4 h before staining. The pellets were dissociated to single cells with TrypLE, washed with PBS0 and consequently stained for FC analysis as described in the flow cytometry section. For the Gal-9 blocking assays, 10 μg/mL anti-Gal-9 mAb (BioLegend) was added to the co-culture from the start of the assay.

#### Immunofluorescent stainings

For the γH2AX-staining of organoids, treated organoids were harvested, washed in PBS0 and fixated in formalin 4%/eosin 0.1% for 1 h at room temperature and transferred to 70% ethanol. Consequently, the organoids were embedded in agarose (2.5%, Eurogentec), processed (Leica ASP 300 S) and embedded in paraffin (Surgipath Paraplast, Leica). The FFPE organoids were sectioned at 4 μm, dried at 55°C overnight and then deparaffinized in xylene and rehydrated in decreasing concentrations of alcohol (by using the Leica autostainer). For antigen retrieval, the slides were incubated in sodium citrate buffer (10 mM, pH 6, Merck) and washed with PBS/Tween 20 (PBST). The slides were blocked with normal goat serum (10% in PBST) for 30 min and incubated with rabbit anti-phospho-histone H2A.X (Ser139) (20E3) (1:200, Cell Signaling) overnight at 4°C. The slides were washed with PBST and then incubated with goat anti-rabbit Alexa 647 (1:200, Thermo Fisher) for 1 h at RT. After additional washing, the slides were mounted with fluoroshield with DAPI mounting medium (Sigma Aldrich).

#### Imaging of organoids

Bright field (co-culture) images were acquired using an EVOS FL Cell Imaging System (Thermo Fisher Scientific). Fluorescence images were generated with a Leica (Wetzlar, Germany) SP8X laser-scanning confocal microscope.

#### Quantification of γH2AX-staining

For the quantification of γH2AX-staining in organoids, images acquired by the confocal microscope were processed in Fiji (ImageJ 1.53q) using a script.^82^ In short, the channels of the RGB images were split, nuclei were defined based on DAPI staining and maxima in the γH2AX channel were measured and quantified per nucleus. The number of foci per nucleus has then been analyzed.

#### CaspaseGlo assay

Organoids were cultured in 96-well plates and treated with indicated conditions for 48 h. Subsequently, the Matrigel was dissolved with GF- and the samples were transferred to a white opaque 96-well plate. Caspase-Glo 3/7 Reagent (Promega) was prepared according to manufacturer’s indications and added to the organoids in a 1:1 ratio to GF- up to a total volume of 100μL. The assay was incubated for 40 min and luminescence was measured with a TriStar2 Multimode plate reader LB942 (Berthold Technologies).

#### Flow cytometry

T cells were stained with live/dead marker Zombie NIR (Biolegend) in PBS0 and then stained with directly conjugated antibodies anti-CD3-PE and anti-CD4^−^or anti-CD8-FITC (BioLegend) either in FACS buffer (PBS0, 2 mM EDTA, 0.5% BSA, Sigma) or MACS buffer. For assessing T cell activation, anti-CD25-APC (BioLegend), anti-CD69-BV605 (BioLegend) were added to the staining mix. Intracellular IFNγ-staining was performed using the Intracellular Fixation & Permeabilization Buffer Set (eBioscience) with anti-IFNg-PECy7 (BD Biosciences). For analysis of proliferation T cells were stained before start of the assay with CTV (Invitrogen, 5 μM in PBS0) according to manufacturer’s protocol. A Dead Cell Apoptosis Kit with Annexin V-FITC and propidium iodide was used according to manufacturer for Annexin V FACS staining. FC data were acquired with a BD LSRFortessa Cell Analyzer (BD Biosciences) using FACSDiva (BD Biosciences) software and a CytoFLEX Flow Cytometer (Beckman Coulter) with CytExpert software. The data were analyzed with FlowJo (Treestar, 10.6.2) or CytExpert software (2.4).

#### CellTraceViolet proliferation assay

For evaluation of the effect of chemotherapy treatment on organoid proliferation, organoids were dissociated into single cells and stained with CTV before plating. After 5 days of culture, organoids were harvested, processed into single cells, stained with Zombie NIR, and analyzed by FC.

#### Mitochondrial damage assay

For the quantification of mitochondrial damage in chemotherapy treated organoids, organoids were incubated with MitoTracker Green FM (100 nM, Thermo Fisher) and Tetramethylrhodamine methyl ester perchlorate (TMRM) (150 nM, Thermo Fisher) for 45 min at 37°C following chemotherapy treatment. After incubation organoids were dissociated into single cells, stained with Zombie NIR and analyzed by FC.

#### Guide RNA-mediated gene knock-out in organoids

Organoids were dissociated in single cells as described above. Single cell organoids were prepared for electroporation (EP) with the Neon Transfection System according to manufacturer’s indications (Thermo Fisher) for CRISPR-Cas9-mediated knock-out (KO) using ribonucleoprotein (RNP) complexes. In short, 1.8 μM crRNA:tracrRNA (guide RNA, gRNA), 1.5 μM Cas9 nuclease, and 1.8 μM Neon Electroporation Enhancer were combined with single cell organoids at a final density of 6000 single organoid cells/μL. Cells were pulsed once with a voltage of 1700 for 20 m. The gRNA sequence used for editing *LGALS9* was: TTTGCTGTGAACTTTCAGAC (PAM: TGG) (predesigned by and purchased from IDT). Organoids were immediately replated after electroporation at a density of 6000 single organoid cells/drop in 50% Matrigel and cultured in hSI-EM supplemented with Rock inhibitor for the first 2 days. A week after electroporation, single organoids were collected and replated for clonal selection. Organoids have been kept in culture for expansion for several weeks in hSI-EM supplemented with primocin to prevent infections. Organoids were lysed to extract protein and validate the KO line by western blot at indicated timepoints.

#### Western blot

For each condition two wells of organoids were collected. After removal of Matrigel by centrifugation (300 g 5 min 4°C), organoids were lysed in RIPA buffer (1% Triton X-100, 1% sodium deoxycholate, 0.1% sodium dodecyl sulfate [SDS], 0.15M NaCl, 0.01M sodium phosphate, pH 7.2) containing HALT protease inhibitor 1× (Thermo Fisher). Protein concentration was determined using Lowry protein assay. Equal amounts of sample (5 μg) were analyzed by SDS polyacrylamide gel electrophoresis (SDS-PAGE) and transferred to polyvinylidene difluoride membrane using the Trans-Blot Turbo System (Bio-rad). The membrane was blocked with 5% milk protein in TBST (0.3% Tween, 10 mM Tris pH 8.5 and 150 mM NaCl in H_2_O) and probed with primary antibodies: anti-Gal-9 (abcam) and anti-β-Actin (Santa Cruz). After washing with TBST, the membrane was incubated with peroxidase (HRP)-conjugated polyclonal secondary antibodies: anti-rabbit (Agilent, for Gal-9) and anti-mouse (Agilent, for β-Actin). The membrane was subsequently washed with PBS0 and detection was done using either SuperSignal West Femto Extended Duration Substrate (Thermo Fisher) (for Gal-9) or SuperSignal West Dura Extended Duration Substrate (Thermo Fisher) (for β-Actin) with a Bio-Rad Chemidoc Touch Imaging system. The membrane was stripped using Stripping buffer (0.4% SDS 200 mM Glycin) before probing with another primary antibody.

#### Luminex

Biobanked plasma samples from HSCT patients at specific time points related to their HSCT date were used for Luminex analysis. The data were collected after patients provided written informed consent (HSCT Biobank, local IRB approval 05–143 and 11-063k) in accordance with the Helsinki Declaration. Plasma had been stored at −80°C until analysis. With Luminex multiplex immunoassay technology, a total of 60 plasma proteins were measured: Gal-9, IL1RA, IL2, IL3, IL4, IL5, IL6, IL7, IL10, IL15, IL17, IL18, IL22, TNFα, IFNα, IFNγ, APRIL, OSM, LAG3, Follistatin, I309, MIP1a, MIP1b, IL8, MIG, IP10, BLC, OPG, OPN, G-CSF, M-CSF, GM-CSF, SCF, HGF, EGF, AR, VEGF, CD40L, sPD1, FASL, IL1R1, IL1R2, ST2, TNFR1, TNFR2, sIL2Rα, sCD27, IL7Rα, sSCFR, Elastase, S100A8, Ang1, Ang2, LAP, TPO, sICAM, sVCAM, MMP3, Gal-3, C5a. The multiplex immunoassay was performed according to the protocol from the MultiPlex Core Facility of the UMCU.^84^

#### RNA sequencing

For RNA-seq of treated organoids, mRNA was isolated using Poly(A) Beads (NEXTflex). Sequencing libraries were prepared using the Rapid Directional RNA-Seq Kit (NEXTflex) and sequenced on a NextSeq500 (Illumina) to produce 75 base long reads (Utrecht DNA Sequencing Facility). Sequencing reads were mapped against the reference genome (hg19 assembly, NCBI37) using the BWA41 package (mem –t 7 –c 100 –M –R).^85^ RNA sequencing was analyzed using DESeq2^86^ in the R2: Genomics Analysis and Visualization Platform (http://r2.amc.nl). A principal component analysis (PCA) was performed, and a list of differentially expressed genes (padj<0.1) was generated. Gene Ontology (GO) term analysis was done using either upregulated (logFoldChange>0) or downregulated (logFoldChange<0) DEGs as compared to control per condition using 2 × 2 contingency table analysis chi-square with continuity correction (padj<0.1). Gene Set Enrichment Analysis (GSEA) Pre-ranked analysis was performed with the GSEA software probing for enrichment of genes belonging to Hallmark datasets in the GSEA software.^83^

The dataset analyzed in this manuscript is uploaded on EGA (EGA: EGAS00001007550).

#### Olink proximity extension proteomic analyses

Organoids were cultured in 24-well plates and treated for 48 h with indicated conditions. After treatment, the medium was refreshed with hSI-EM without p38 inhibitor for 24 h. Consequently, the CM was harvested and centrifuged 1000 *g* for 5 min. The supernatant was transferred to new Eppendorf tubes and stored at −80°C until analysis. The Olink Target 96 Immuno-Oncology panel (v.3112) from Olink (Uppsala, Sweden) was used to quantify 92 immuno-oncology related proteins in each sample (IL-8, TNFRSF9, TIE2, MCP-3, CD40-L, IL-1 alpha, CD244, EGF, ANGPT1, IL-7, PGF, IL-6, ADGRG1, MCP-1, CRTAM, CXCL11, MCP-4, TRAIL, FGF2, CXCL9, CD8A, CAIX, MUC-16, ADA, CD4, NOS3, IL-2, Gal-9, VEGFR-2, CD40, IL-18, GZMH, KIR3DL1, LAP TGF-beta-1, CXCL1, TNFSF14, IL-33, TWEAK, PDGF subunit B, PDCD1, FASLG, CD28, CCL19, MCP-2, CCL4, IL-15, Gal-1, PD-L1, CD27, CXCL5, IL-5, HGF, GZMA, HO-1, CXCL1, CXCL10, CD70, IL-10, TNFRSF12A, CCL23, CD5, CCL3, MMP7, ARG1, NCR1, DCN, TNFRSF21, TNFRSF4, MIC-A/B, CCL17, ANGPT2, PTN, CXCL12, IFN-gamma, LAMP3, CASP-8, ICOSLG, MMP12, CXCL13, PD-L2, VEGFA, IL-4, LAG3, IL12RB1, IL-13, CCL20, TNF, KLRD1, GZMB, CD83, IL-12, CSF-1). Multiplex proximity extension assay panels were used to quantify each protein, as previously described.^51^ The raw quantification cycle values were normalized and converted into normalized protein expression (NPX) units. The NPX values were expressed on a log2 scale in which one unit higher in NPX values represents a doubling of the measured protein concentration. Quality control of the measured samples was performed by using the standard quality control protocol of Olink.

#### qRT-PCR

RNA was isolated from organoids after treatment and stored at −80°C. Reverse transcriptase quantitative PCR (RT-PCR) was performed with a iScript cDNA Synthesis Kit (BioRad). cDNA (20–50 ng) was amplified with TaqMan Fast Advanced Master Mix for qPCR (Applied Biosystems) according to the manufacturer instructions in a CFX96TM Real-Time PCR Detection System (BioRad). Relative amounts of mRNA were calculated by the comparative ΔCt method with *HP1BP3* as housekeeping gene. The following TaqMan probes were used: *GADD45A* (ID: Hs00169255_m1), *HP1BP3* (ID: Hs00212448_m1), *LGALS9* (ID: Hs00371321_m1), MDM2 (ID: Hs00540450_s1), *PARP2* (ID: Hs00193931_m1).

### Quantification and statistical analysis

Data are presented as mean ± standard error of the mean (SEM). To take into account intra-individual and intra-experimental variation experiments were performed at least twice with several wells per condition, and sample material coming from at least two different human donors. Statistical significance was determined at *p ≤* 0.05 using an ordinary or row-matched (RM) 1-way analysis of variance (ANOVA) with Dunnett’s multiple comparison test, paired or unpaired *t* test, or multiple *t* tests with false discovery rate correction for multiple comparisons where appropriate, using Prism 9 software (GraphPad). Significance is indicated as *p ≤* 0.05 (∗), *p ≤* 0.01 (∗∗), or *p ≤* 0.001 (∗∗∗) or *p* < 0.0001 (∗∗∗∗).

## Supplemental information

Document S1. Figures S1–S4

Table S1. RNA-seq analysis of chemotherapy-damaged organoids, related to Figure 2
