# Supplementary material for: Chemotherapy-induced intestinal epithelial damage directly promotes galectin-9-driven modulation of T cell behavior
Source: iScience. 2024 May 22;27(6):110072. doi: 10.1016/j.isci.2024.110072 (PMC11176658; doi:10.1016/j.isci.2024.110072)
Supplement: Document S1. Figures S1–S4 [file mmc1.pdf]

iScience, Volume *27*

Supplemental information

Chemotherapy-induced intestinal epithelial damage directly promotes galectin-9-driven modulation of T cell behavior

Suze A. Jansen, Alessandro Cutilli, Coco de Koning, Marliek van Hoesel, Cynthia L. Frederiks, Leire Saiz Sierra, Stefan Nierkens, Michal Mokry, Edward E.S. Nieuwenhuis, Alan M. Hanash, Enric Mocholi, Paul J. Coffer, and Caroline A. Lindemans

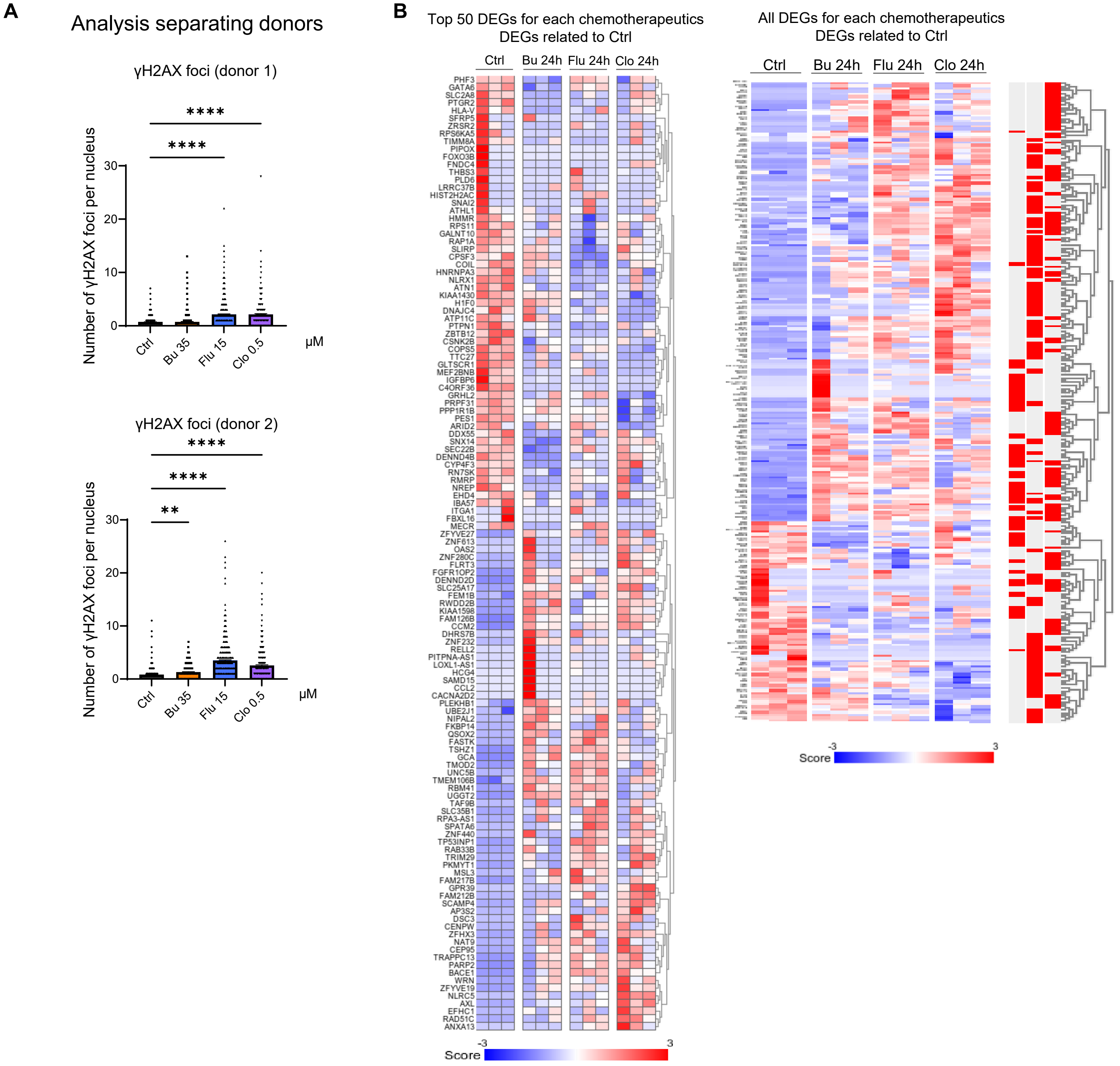

Figure S1. Additional characterization of chemotherapy-induced damage of intestinal organoids. Related to Figure 1 and Figure 2. (A) Quantification of phosphorylated (γ) histone (H)2AX (γH2AX) foci per nucleus in different organoid donors, mean with SEM, ANOVA **(B)** Heatmaps of a combined list made from the top 50 DEGs of 24 hour busulfan-, fludarabine- and clofarabine-treated organoids versus control (left) or all DEGs (right). Minimal number of samples containing a present call was set to 2. Significance is indicated as $P \leq 0.05$ (*), $P \leq 0.01$ (**), or $P \leq 0.001$ (***) or $P < 0.0001$ (****).

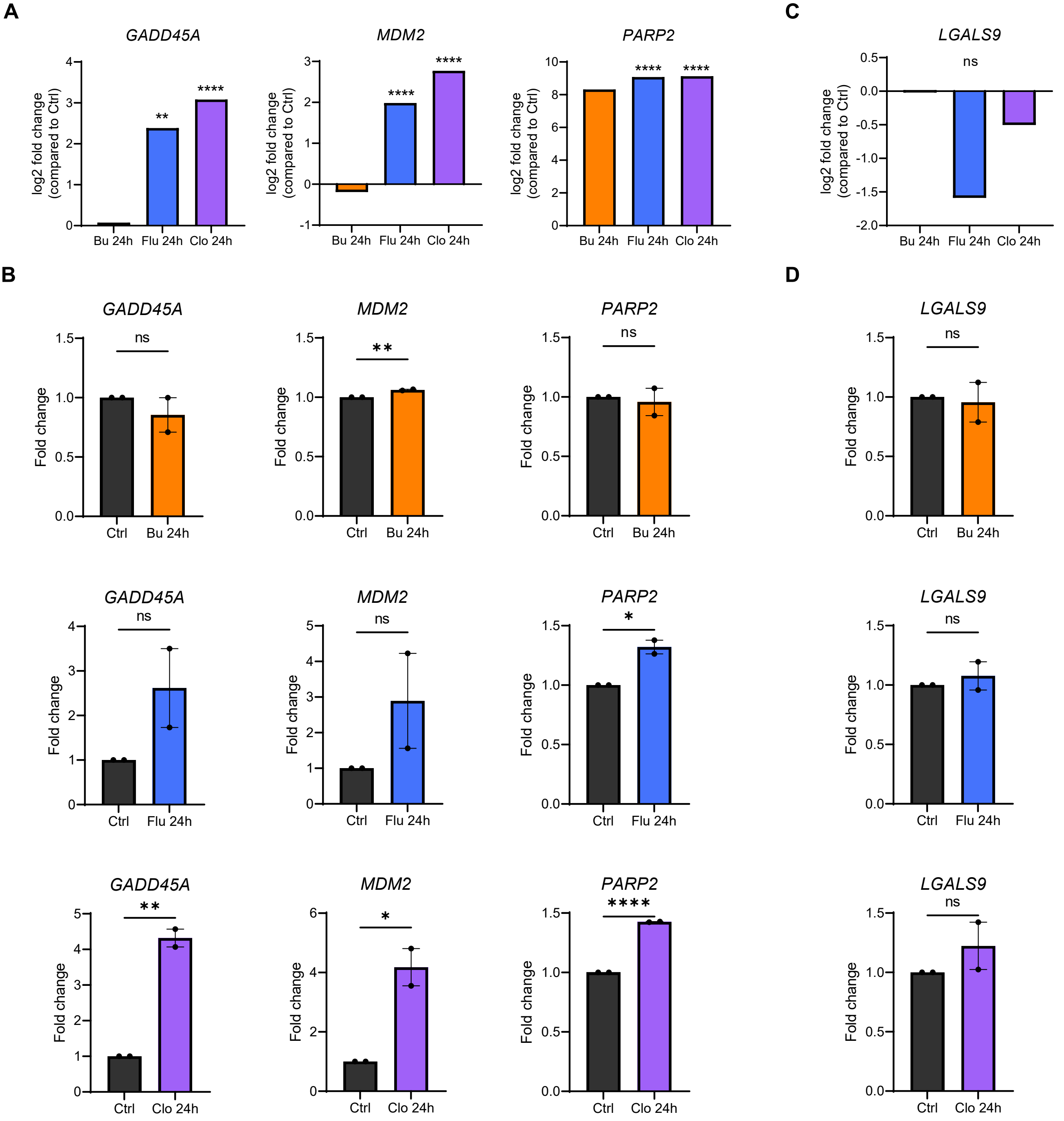

Figure S2. Validation of RNA-seq of chemotherapy-damaged intestinal organoids. Related to Figure 2. Comparison between RNA-seq and qPCR of chemotherapy-treated intestinal organoids. Intestinal organoids from different donor have busulfan (Bu, 3.5μM), fludarabine (Flu, 15μM), clofarabine (Clo, 0.5μM) for 24h. **(A)** Expression of *GADD45A*, *MDM2*, and *PARP2* extrapolated from Table S1 (n=3 donors). DESeq2. **(B)** Expression of *GADD45A*, *MDM2*, and *PARP2* by qPCR (n=2 donors). Mean with SEM, unpaired *t* test. **(C)** Expression of *LGALS9* extrapolated from Table S1 (n=3 donors). DESeq2. **(D)** Expression of *LGALS9* by qPCR (n=2 donors). Mean with SEM, unpaired *t* test. Significance is indicated as $P \leq 0.05$ (*), $P \leq 0.01$ (**), or $P \leq 0.001$ (***) or $P < 0.0001$ (****).

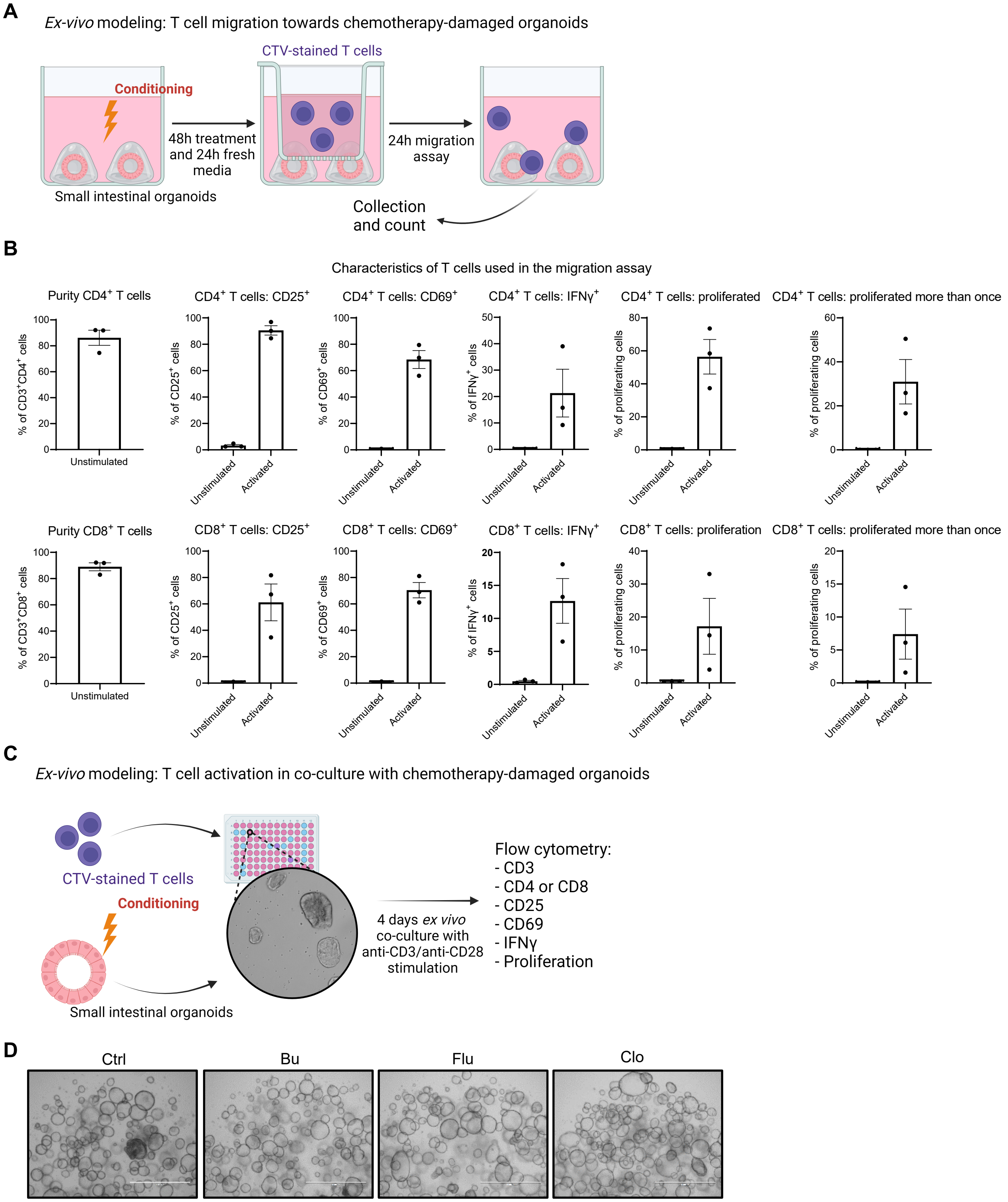

Figure S3. Additional details on migration and co-culture assays. Related to Figure 3. (A) Schematic overview of the migration assay. Illustration made with BioRender.com. **(B)** Purity, activation markers, and proliferation of CD4+ and CD8+ T cells polyclonally activated for 3 days before start of the migration assay. **(C)** Schematic overview of the co-culture system to evaluate T cell activation by polyclonal stimulation in presence of organoids. Illustration made with BioRender.com. **(D)** Representative EVOS images of organoids treated for 24h with busulfan (35μM), fludarabine (15μM), clofarabine (0.5μM). Images are taken before the start of the co-culture assay, scale bar = 1000 μm. Significance is indicated as P ≤ 0.05 (*), P ≤ 0.01 (**), or P ≤ 0.001 (***) or P < 0.0001 (****).

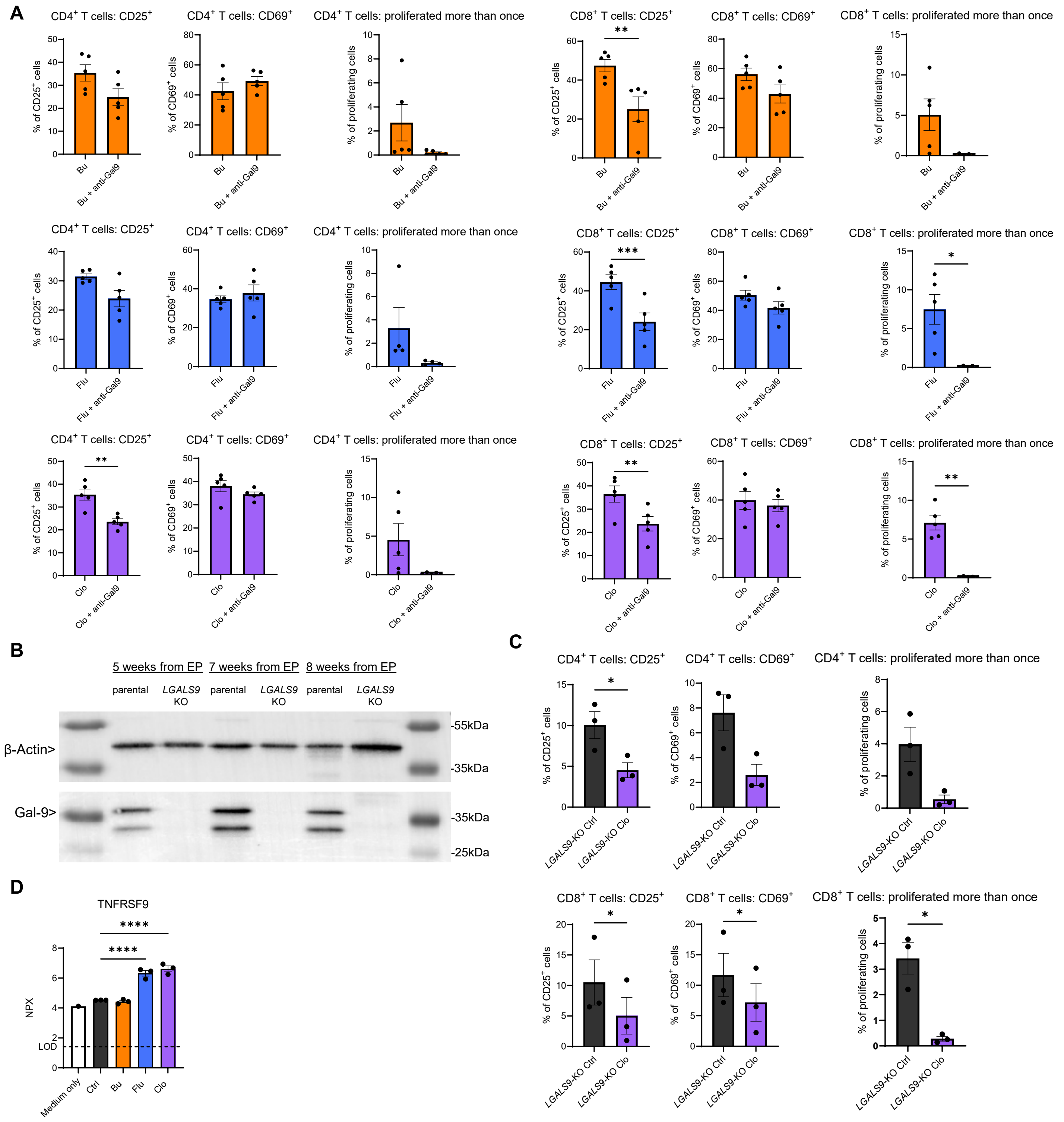

Figure S4. Additional details T cell assays with neutralizing antibody anti-Gal-9 and *LGALS9*-knock-out organoids. Related to Figure 4. (A) $CD4^+$ and $CD8^+$ T cell activation after co-culture with treated organoids in the presence of anti-galectin-9 (Gal-9) monoclonal antibody. N≥5 T cell donors with 1 organoid donor, each data point indicates a T cell donor, mean with SEM, paired *t* test. **(B)** Western blot with protein lysates from organoids after electroporation (EP) with a CRISPR-Cas9 ribonucleoprotein complex (RNP) targeting *LGALS9*. KO: knock-out **(C)** $CD4^+$ and $CD8^+$ T cell activation and proliferation after co-culture with *LGALS9*-KO organoids. N=3 T cell donors with 1 organoid donor, each data point indicates a T cell donor, mean with SEM, paired *t* test. **(D)** Soluble-TNFRSF9 levels detected by Olink proteomics. Each data point indicates the levels of TNFRSF9 in the conditioned medium from each organoid condition (log scale) as normalized protein expression (NPX) units, mean with SEM, ANOVA. LOD: level of detection. Significance is indicated as P ≤ 0.05 (*), P ≤ 0.01 (**), or P ≤ 0.001 (***) or P < 0.0001 (****).
